# EHMTI-0150. A controlled study of sleep in cluster headache patients.apo04

**DOI:** 10.1186/1129-2377-15-S1-E13

**Published:** 2014-09-18

**Authors:** M Barloese, P Jennum, N Lund, R Jensen

**Affiliations:** 1 Neurology, Danish Headache Center, Glostrup, Denmark; 2 Neurology, Danish Center for Sleep Medicine, Glostrup, Denmark

## Background

Cluster headache (CH) is a primary headache disorder characterized by notoriously severe attacks of unilateral pain following a chronobiological pattern. There is a close connection with sleep as many attacks occur during the night and a complex hypothalamic involvement has been suggested.

## Aim

To investigate sleep in a large, well-characterized population of CH-patients and compare our findings to those in healthy controls.

## Methods

We performed polysomnography (PSG) for two nights in 40 CH patients during active bout and for one night in 25 age, sex and BMI-matched controls on an in-hospital basis. Clinical headache characterization was obtained through a semi-structured interview.

## Results

A total of 99 nights of PSG were analyzed. Our main finding was a reduced percentage of REM-sleep (P<0.01), longer REM-latency (P<0.01) and fewer arousals (P<0.01) in CH patients. There was no difference in the prevalence of sleep apnea between patients (38%) and our matched controls (32%) although numerically patients had a higher mean apnea-hypopnea index (10.75 vs. 4.93). We observed 45 nocturnal CH attacks but no temporal association with particular sleep stages.

## Conclusions

To date, this is the largest study of sleep in CH. REM-sleep is affected in CH which is in line with our current understanding of CH and newer studies indicating hypothalamic involvement in the regulation of this sleep stage. Further, we found fewer arousals in CH-patients as has been demonstrated in other headache disorders. Together, the findings support a central role of the hypothalamus and arousal systems in CH.

No conflict of interest.

